# Nasopharyngeal Swabs for *Orientia tsutsugamushi* Detection in Doxycycline Treatment: A Prospective Cohort Study in Hainan, China

**DOI:** 10.3390/pathogens15020158

**Published:** 2026-02-02

**Authors:** Yuanze Chen, Siqi Chen, Jiajia An, Xiaojing Zheng, Qi Wang, Yuyan Wang, Wenjing Fu, Biao Wu, Yongguo Du, Feifei Yin, Liyuan Zhang

**Affiliations:** 1 Department of Infectious Diseases, The Second Affiliated Hospital, Hainan Medical University, Haikou 570311, China; cyuanze@muhn.edu.cn (Y.C.); 17754998135@muhn.edu.cn (J.A.); 15120852285@163.com (X.Z.); wq518@muhn.edu.cn (Q.W.); 15857075574@163.com (Y.W.); 18688148093@muhn.edu.cn (W.F.); duyongguo@163.com (Y.D.); 2 The University of Hong Kong Joint Laboratory of Tropical Infectious Diseases—Hainan Medical University, Key Laboratory of Tropical Translational Medicine of Ministry of Education, Academician Workstation of Hainan Province, School of Basic Medicine and Life Sciences, Hainan Medical University, Haikou 571199, China; qisi49u@muhn.edu.cn; 3 Hainan General Hospital, Hainan Affiliated Hospital of Hainan Medical University, Haikou 570100, China; wubiao@hainmc.edu.cn

**Keywords:** scrub typhus, nasopharyngeal swabs (NPSs), urine, stool, qPCR, sanger sequence

## Abstract

Scrub typhus, caused by *Orientia tsutsugamushi*, remains a neglected cause of acute febrile illness. Molecular testing of blood supports early diagnosis, yet once doxycycline is started, blood qPCR positivity can drop rapidly, complicating short-term follow-up and relapse surveillance. We compared detection across multiple clinical specimens and evaluated nasopharyngeal swabs (NPSs) as noninvasive supplementary specimens during treatment initiation. In a prospective single-center cohort from Hainan, China, we enrolled 20 patients with scrub typhus. Blood, NPS, urine, and stool were collected before doxycycline administration 24 h after the first dose and on day 5. qPCR was performed for the analysis of *Orientia tsutsugamushi*. qPCR-positive specimens were subjected to nested PCR targeting TSA56, and nested PCR-positive amplicons were Sanger sequenced for genotyping. Before treatment, *O. tsutsugamushi* DNA was detected in 15/20 blood samples (75.00%) and 5/20 NPS samples (25.00%), but 0/20 urine samples (0%) and 0/20 stool samples (0%). At 24 h after treatment, detection in blood was 0/20 (0%) while NPS samples were positive in 3/20 (15.00%). All specimens were negative by day 5 after treatment. Across sequenced NPS positives (*n* = 3), *Karp* 2/3 (66.77%) and *Gilliam* 1/3 (33.33%) predominated. In paired blood–NPS positives, inter-specimen homology was high (percentage nucleotide identity 100% for *Karp* and 100% for *Gilliam*). NPS is not sensitive enough for primary diagnosis; however, within the first 24 h after doxycycline it offers a practical, noninvasive supplementary specimen to support short-term follow-up and community-based sampling when venipuncture or transport are constrained. Larger, multi-center studies are warranted to refine sampling windows and diagnostic performance.

## 1. Introduction

Scrub typhus, also known as tsutsugamushi disease, is an acute natural focal infectious disease caused by *Orientia tsutsugamushi* (*O. tsutsugamushi* (OT)) and transmitted to humans by the bites of infected chigger mites. The annual incidence in the Asia–Pacific region is approximately 1 million cases, and the fatality rate of untreated cases can reach 30% [1,2]. Its distribution spans from Japan in the east, Pakistan in the west, Russia in the north, and Australia in the south, in a region known as the “Tsutsugamushi Triangle” [3]. However, in recent years, its geographic range has expanded, posing a growing public health concern [3,4,5,6].

OT is an obligate intracellular pathogen. In 2002, after OT was first detected in eschar in Japan [7], PCR testing has been primarily used for blood and eschar samples and its detection results can aid in disease diagnosis. However, the detection rate in blood samples is strongly affected by antimicrobial agents. At 12–24 h after doxycycline administration, pathogen nucleic acid in blood rapidly becomes undetectable, which poses significant challenges for efficacy evaluation, relapse monitoring, and epidemiological investigations.

The study of the pathophysiology of tsutsugamushi has indicated that OT may be extensively distributed in the human body. The nasopharynx is an accessible mucosal surface involved in the early interaction of many pathogens with the host; therefore, nasopharyngeal swabs (NPSs) may provide noninvasive supplementary specimens for OT detection, particularly during treatment initiation. In this prospective cohort, 20 scrub typhus patients were enrolled at the Second Affiliated Hospital of Hainan Medical University between June 2024 and June 2025. *O. tsutsugamushi* detection in blood, NPS, urine, and stool was compared before doxycycline, 24 h after the first dose, and on day 5. TSA56 genotyping and phylogenetic analysis were performed on positive samples to characterize detected strains and to evaluate the feasibility of NPS for short-term follow-up after doxycycline.

## 2. Materials and Methods

### 2.1. Ethics Committee

This prospective cohort study was approved by the Ethics Committee of the Second Affiliated Hospital of Hainan Medical University. Ethical approval was granted under the reference number LW-2022-033 (approved on 31 March 2022) and remained valid through the committee’s required annual continuing review. Written informed consent to participate in this study was obtained from all the patients.

### 2.2. Study Population, Diagnostic Criteria, and Data Collection

Clinical samples and clinical data of patients diagnosed with scrub typhus at the Second Affiliated Hospital of Hainan Medical University between June 2024 and June 2025 were collected. The classification for suspected, clinically diagnosed, and confirmed cases was based on the Expert consensus on clinical diagnosis and treatment of scrub typhus (2024 edition) [8]. A summary of the case definitions is available in Supplementary Materials S1 Text. All patients enrolled in this study were confirmed cases.

For all enrolled patients, a comprehensive dataset was compiled, including general demographic and clinical information (sex, age, initial symptoms, prior antibiotic use, complications, comorbidities, and underlying diseases), results of routine laboratory tests (complete blood count, liver and kidney function panels, and coagulation function assays), and findings from imaging examinations (chest computed tomography (CT) and abdominal color Doppler ultrasound).

### 2.3. Sample Collection

For all scrub typhus patients, blood, urine, stool, and NPS specimens were obtained at three key time points: pre-doxycycline (at admission), 24 h after the first doxycycline dose, and on day 5 of doxycycline therapy. To ensure sample quality, blood, urine, stool, and NPS samples were stored at 4 °C and transported to the laboratory in a sealed refrigerated transport box (2–8 °C). Nucleic acid extraction was initiated immediately upon arrival and completed within 48 h of collection.

### 2.4. Sample Processing and DNA Extraction

DNA was extracted from whole blood, nasopharyngeal swabs (NPSs), urine, and stool using an automated nucleic acid extraction system with the CS-B200 extraction kit (Zybio Inc., Chongqing, China), following the manufacturer’s standardized procedures for complex clinical specimens, including respiratory matrices. Whole blood was collected into EDTA anticoagulant tubes and aliquoted prior to nucleic acid extraction.

Pre-analytical processing was performed as follows: (i) whole blood: 200 μL, was directly processed for extraction; (ii) NPS: swabs were placed in a specimen preservation medium and 1 mL of the medium was centrifuged at high speed; the supernatant was discarded, the pellet was resuspended, and 200 μL of the resuspended pellet was used for extraction; (iii) urine: specimens were centrifuged at high speed and 200 μL of the resuspended pellet was used for extraction; (iv) stool: specimens were suspended in normal saline, vigorously vortexed, and 200 μL of the homogenized suspension was used for extraction. DNA was eluted in a final volume of 60 μL.

### 2.5. PCR Detection and Sequence Analysis of Orientia tsutsugamushi

A real-time fluorescent quantitative PCR (qPCR) assay and a nested PCR protocol were implemented based on the 56-kDa type-specific antigen gene (TSA56) of *O. tsutsugamushi*. Primer/probe design was guided by representative TSA56 sequences (GenBank accession nos. AY357216, AF050669, AY222631, AY222635, and M33004) and previous publications. A two-step molecular workflow was used for screening and genotyping.

First, DNA extracts were screened using an *O. tsutsugamushi* Nucleic Acid Detection Kit (Zybio Inc., Chongqing, China) targeting species-specific loci, including the 47-kDa, 56-kDa, and groEL genes; commercial quality control materials supplied for the assay were used as the negative and positive controls and testing was performed according to the manufacturer’s instructions. All samples were tested in replicate reactions. A sample was considered OT positive when all three target loci showed typical amplification curves with Ct < 40 in the replicate reactions and the internal/process control met the predefined acceptance criteria. Borderline (near-threshold) or discordant results between replicates were retested. Second, TSA56 was measured separately for quantitative detection of *O. tsutsugamushi* using a singleplex TaqMan qPCR assay (TaKaRa Bio Inc., Otsu, Shiga, Japan). A recombinant plasmid containing a TSA56 gene fragment served as the positive control and quantification standard. The TSA56 primers and hydrolysis probe were: forward 5′-TGATAAGGATATTAAAGGGCATA-3′, reverse 5′-ATACACCCTCAGCAGCATTAAT-3′, and probe 5′-(FAM)-ATGGTTGCATCAGGAGCACTTGG-(BHQ1)-3′.

The nested PCR targeting TSA56 was performed to generate an expected 483 bp amplicon. The first-round PCR used forward 5′-TCAAGCTTATTGCTAGTGCAATGTCTGC-3′ and reverse 5′-AGGGATCCCTGCTGCTGTGCTTGCTGCG-3′. The second-round PCR used forward 5′-GATCAAGCTTCCTCAGCCTACTATAATGCC-3′ and reverse 5′-CTAGGGATCCCGACAGATGCACTATTAGGC-3′. Nucleic acid extracted from an *O. tsutsugamushi*-positive clinical specimen was included as the positive control, and a no-template control was included in each run to monitor potential contamination. Amplicons were purified and subjected to bidirectional Sanger sequencing for downstream phylogenetic analyses. Nested PCR amplicons were purified and submitted to Sangon Biotech (Shanghai, China) for bidirectional Sanger sequencing on an ABI 3730xl DNA Analyzer (Applied Biosystems, Foster City, CA, USA). Forward and reverse reads from the same specimen were assembled and manually checked using SeqMan (DNASTAR Lasergene v18.1.1; Madison, WI, USA) to generate a confirmed consensus sequence for downstream analyses. The obtained sequences were deposited in GenBank (accession nos. PX714883–PX714900). Closest matches were identified by BLAST v2.17.0 (National Center for Biotechnology Information, Bethesda, MD, USA) against the NCBI database, and alignments were generated using CLUSTALX v2.1 (European Bioinformatics Institute, Hinxton, UK) with the closest related and representative global reference sequences. Phylogenetic trees were reconstructed using the neighbor-joining method with 1000 bootstrap replicates.

### 2.6. Data Analysis

R4.2.2 software was used for statistical analysis. Measurement data that conformed to a normal distribution were analyzed using the independent sample *t* test and are described as ($\bar{x}$ ± SD). The Mann–Whitney U-rank sum test was used, and the median and interquartile range [M (Q1, Q3)] were used to describe the data. Count data are expressed as the number of cases or percentage.

## 3. Results

### 3.1. General Information

The 20 patients included 12 males and 8 females, aged 58.00 ± 10.31 years. Five patients had a clear history of field activities. Among the 14 patients who sought medical care after symptom onset, visits were made to secondary medical institutions (namely, seven Class II Grade A hospitals, three community hospitals, and four local clinics), but none received a definitive diagnosis. All the patients were admitted to the hospital with fever as the first symptom. After admission, they were given doxycycline for infection control, an antipyretic, supplemental oxygen, and supportive therapy for liver and kidney protection. The general information is shown in Table 1.

### 3.2. Analysis of Clinical Characteristics

All patients (100%) presented with fever as the first symptom, and the median duration of fever was 7 (5, 8.50) days. The main concomitant symptoms included dizziness, headache (13 cases; 65%), and chills (11 cases; 55%). Laboratory tests revealed abnormal liver and kidney function, changes in white blood cell and platelet counts, and abnormal coagulation. Some patients presented multisystem involvement, combination with underlying diseases, and multiple pathogen infections.

During physical examination, five patients (25%) presented with characteristic eschar, and the distribution locations included the axilla (three patients), groin (one patient), and scrotum (one patient). Four patients (20%) presented with splenomegaly, and one patient presented with a systemic rash (5%). Chest CT imaging examination revealed that 12 patients (60%) presented multiple inflammatory/exudative lesions in both lungs. Among these, three patients (15%) had small amounts of pleural effusion. Additionally, lymphadenopathy, predominantly in the axillary region, was found in three patients (15%). Moreover, three cases (15%) were associated with central nervous system abnormalities (such as lacunar infarction), and two patients (10%) had cardiovascular abnormalities (all with newly developed atrial premature beats, Table 1).

### 3.3. Laboratory Test Results

A total of 60 blood samples, 60 NPS samples, 60 urine samples, and 20 stool samples were collected from 20 patients with scrub typhus at different time points (Table 2). Because bowel habits vary among patients and are influenced by illness and food intake, collecting stool specimens at every scheduled time point was not feasible; therefore, stool was collected only at the first bowel movement after admission for each patient. All samples were first screened by qPCR. qPCR-positive samples were then tested by nested PCR targeting the 56-kDa TSA gene, and Sanger sequencing was performed for nested PCR-positive amplicons.

Among the 20 scrub typhus patients, 15/20 (75.00%) blood specimens before doxycycline were positive by qPCR, but all negative at 24 h after treatment and 5 days. A total of 5/20 (25.00%) NPS samples before doxycycline were positive, versus 3 positive samples at 24 h after treatment (3/20, 15.00%). All qPCR-positive reactions of NPS samples showed typical amplification curves with Ct values < 40, meeting the predefined positivity criteria (Table 3). In total, 15 whole-blood specimens yielded definitive nested PCR amplicons and were successfully sequenced, generating 15 TSA56 sequences. For NPS, 8 specimens yielded definitive nested PCR and only 3 NPS specimens produced sequenceable amplicons, generating 3 TSA56 sequences. All urine (*n* = 60) and stool (*n* = 20) specimens were negative by qPCR (Table 2).

**Figure 1:**
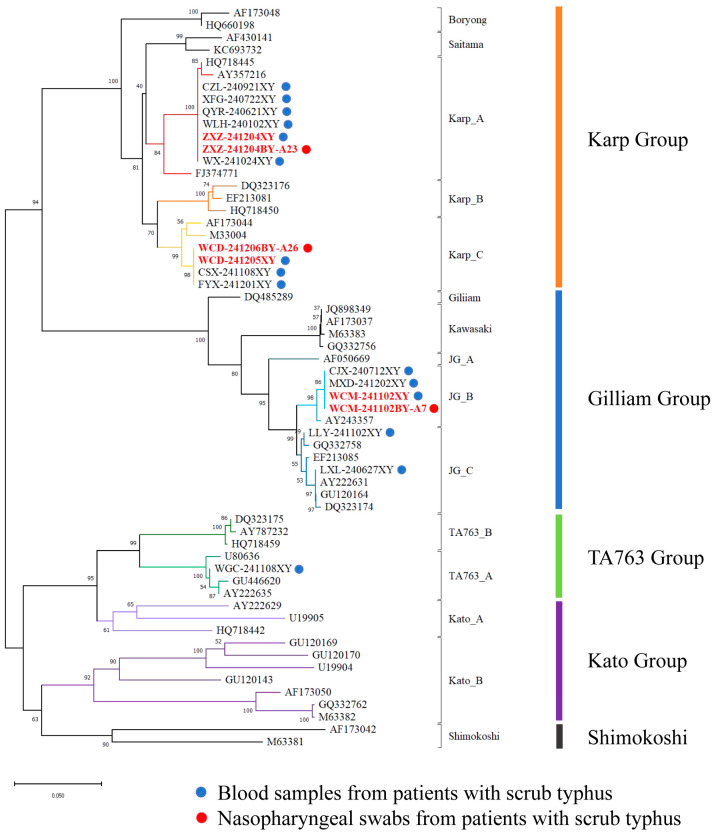
Phylogenetic tree of *Orientia tsutsugamushi* based on TSA56 gene sequences.

Genetic analysis based on TSA56 gene sequences obtained from the 18 nested PCR-positive specimens identified three genotypes, namely *Karp*, *Gilliam*, and *TA763* (Figure 1). Among these 18 positive specimens, paired whole-blood and nasopharyngeal swab (NPS) specimens from three patients were simultaneously positive (six specimens in total) (Table 4). Phylogenetic reconstruction showed that these six sequences clustered into well-supported genotype clades defined by GenBank reference strains, specifically the *Karp_A*, *Karp_C*, and the *Gilliam*-related *JG_B* clusters; moreover, for each of these three patients, the paired blood and NPS sequences were identical at the nucleotide level (100% identity).

The tree was constructed using TSA56 sequences generated in this study together with representative reference sequences retrieved from GenBank. All reference sequences are listed in Supplementary Table S1. Branch colors indicate distinct genotypes (annotated on the right), with major lineages including *Karp*, *Gilliam*, *TA763*, *Kato*, and *Shimokoshi*. Blue dots denote sequences from 15 OT PCR-positive blood samples, and red dots denote sequences from 3 OT PCR-positive nasopharyngeal swabs. Sample IDs in bold red indicate dual-site positivity (OT detected in both blood and nasopharyngeal swab specimens). Numbers at nodes represent bootstrap support values, and the scale bar indicates nucleotide substitutions per site.

## 4. Discussion

This study is the first report on positive nasopharyngeal swabs in OT-infected patients with scrub typhus in Hainan Province. The results of this study are important with regards to the comprehensive testing of multiple samples of stool, urine, blood and nasopharyngeal swabs from clinically diagnosed patients. Using real-time PCR and nested PCR, we successfully detected the nucleic acid of OT in nasopharyngeal swab samples from some patients. Although the positive rate was low, the 100% nucleotide level between the gene sequencing results suggested that the nasopharynx might be a site of transient colonization by OT. These findings challenge the traditional view that OT is limited to the blood or eschar and provide a new perspective for understanding the distribution of OT in the host after infection. Consistent with the findings of previous studies, the *Karp* genotype was dominant in this region [9,10]. Its biological characteristics may affect the distribution of pathogens in different host tissues. The positive nasopharyngeal swab samples were all prior to antibiotic treatment, and the CT values of the samples after treatment significantly increased, suggesting that antibiotic treatment may reduce the sensitivity of PCR detection by reducing the OT load, which is consistent with previous studies demonstrating the established efficacy of doxycycline against scrub typhus [11,12].

The nasopharynx is the primary interface between the respiratory tract and the outside world. The submucosa, which is rich in macrophages and lymphoid tissues, may theoretically become the site for early adhesion, invasion, and even replication of OT. As early as 2003, using Hitachi 12A and Hitachi H-7000 transmission electron microscopes (Tokyo, Japan), Kadosaka et al. [13] reported the accumulation of a large amount of OT in the cytoplasm of the salivary glands of uneaten larvae. In 2014, they were the first to use PCR technology in an animal model and reported OT accumulation in the lungs, spleen, liver, and other organs of the animals. The detection of OT DNA suggested that the lungs could be used as a potential target organ of natural hosts [14]. The mouse model of severe scrub typhus established by Trent B et al. [15] suggested a severe inflammatory response in the lungs; the OT load in the lungs peaked on the tenth day and was significantly higher than that in any other organ (heart, liver, or brain) on the fourteenth day [14] Pathogens can be detected in bronchoalveolar lavage fluid (BALF) [16]. These experiments indirectly indicated that the respiratory tract is no longer a simple “passage channel” but instead an active place for the distribution and replication of pathogens. In this study, we detected OT nucleic acid in nasopharyngeal swab samples before doxycycline administration in some patients. Our results were consistent with those of the abovementioned animal and basic experiments. These findings suggest that OT DNA can be detectable in the nasopharynx during early human infection, though the clinical significance of this detection requires further investigation.

However, we did not detect OT in any of the urine or stool samples, which may be related to its own characteristics. OT is an obligate intracellular parasitic pathogen. Recently, many studies have shown that OT can be detected in endothelial cells, as well as spleen and lung tissues [14,17]. However, no relevant reports of active secretion or shedding into the intestinal cavity or urinary tract have been published. This may explain why the OT load is extremely low in the urine and stool of patients and is difficult to detect by high-sensitivity PCR. Our experimental results are consistent with this conclusion. Through systematic detection of clinical samples using high-sensitivity PCR, we similarly failed to detect OT nucleic acid in patient urine and stool samples. This verifies the theoretical speculation that, due to its obligate intracellular parasitic nature, OT is unlikely to be released from primary infection sites into excretions.

In the OT phylogenetic tree of the TSA56 gene sequence, approximately 66.66% of the OT strains were highly homologous with the *Karp* genotype, which is consistent with the dominant role of this genotype in the global epidemic of scrub typhus [18]. Notably, although this genotype has been reported many times in previous studies, it was detected in nasopharyngeal swab samples for the first time, and its nucleotide level in blood samples from patients was 100%. These findings suggested the genetic stability of this genotype in different host tissues.

As early as 2009, Kelly et al. [18] divided OT into nine major genotypes through the TSA56 gene. Among them, the *Karp* type is the most common, particularly in the Asia–Pacific region, accounting for approximately 40% of all genotypes. The *Karp* type has been detected in numerous locations, such as China (e.g., Hainan, Guangdong, Fujian, Shandong, and the Penghu Islands), the Republic of Korea, Japan, and Southeast Asia (e.g., Vietnam and Cambodia) [18,19] and gradually increased from south to north in India [20]. Kato-type strains, accounting for approximately 10% [18], were the dominant strains in India and were also sporadically found in Vietnam, Cambodia, and Japan. *TA763*, which was first found in Thailand, also accounted for approximately 10% [18]. Subsequently, *TA763* was reported in China, Vietnam, Cambodia, and other places. The *Gilliam* type is the least dominant, accounting for only 5%, and is distributed mainly in Myanmar and northern India [18]. In addition, the *Kawasaki*, *Kuroki*, and *JG* genotypes are prevalent in Japan, whereas the *Boryong* genotype is predominant in the Republic of Korea [18]. Therefore, the Asia–Pacific region showed a distribution pattern dominated by the *Karp* genotype, with the coexistence of multiple genotypes. However, reports on genotypes and their distribution characteristics other than those of the Asia–Pacific region are rare. Therefore, future efforts should focus on expanding the scope of surveillance to address this long-standing knowledge gap.

In China, genotypes significantly differ between the north and south. In southern China, in regions such as Hainan [10], Guangdong [21], and Yunnan [22], the predominant genotypes are *Karp*, *Gilliam*, and *TA763*. In contrast, in northern areas such as Shandong [23] and Inner Mongolia [1], the *Kawasaki* type is dominant. Kim et al. [24] reported that different genotypes may affect the clinical outcomes of patients. In *Karp*-type strains, a relatively high viral load is associated with a high likelihood of organ failure and an increased likelihood of admission to the intensive care unit (ICU). The mechanism may be related to the immune response of the body to pathogens. During OT infection, the body secretes many proinflammatory cytokines, such as IFN-γ, TNF-α, and IL-6, resulting in a “cytokine storm”, causing damage to vascular endothelial cells and multiple organ failure [25]. Similar findings were reported by Jiali Long et al. [21], in which patients infected with *Karp*-type strains showed more frequent multi-organ involvement and worse prognostic indicators. However, whether genotype information can inform risk stratification or genotype-guided management requires validation in larger, prospective studies integrating genotypes with bacterial load, severity, and treatment response

This study has several limitations, such as its small sample size and single-center nature. Nevertheless, studies simultaneously assessing OT in NPS, urine, and feces remain scarce, and the present study may help clinicians better understand its distribution across these specimens. This study provides a new perspective and basis for disease prevention and control. In the future, we should increase the sample size and use multicenter studies to confirm the feasibility and scope of the application of the nasopharyngeal swab test.

## 5. Conclusions

In summary, this study suggested that NPS is not sensitive enough to replace blood (or eschar) for initial diagnosis, but it can serve as a noninvasive, supplementary specimen during the short window after antibiotics are started—useful for follow-up in outpatient or community settings. These pilot data from a single center provide a practical option for clinicians and there is a need for larger studies to define accuracy and timing more precisely.

## Figures and Tables

**Table 1:** Demographic and clinical characteristics of scrub typhus patients.

Clinical Characteristics	Scrub Typhus Patient (*n* = 20)
Age (year)	58.00 ± 10.31
Male (*n*, %)	(12, 60%)
Female (*n*, %)	(8, 40%)
Farmer (*n*, %)	(17, 85%)
Time from the onset of symptoms to admission, days	7 (5, 8.50)
Doxycycline use time, days	7.20 ± 2.00
Inflammatory infiltration/exudation/pleural effusion (*n*, %)	(12, 60%)
Eschar (*n*, %)	(5, 25%)
Rash (*n*, %)	(1, 5%)
Swelling of lymph nodes (*n*, %)	(3, 15%)
Cerebral infarction/new arrhythmia (*n*, %)	(4, 20%)
Hypertension/diabetes mellitus/chronic liver disease (*n*, %)	(5, 25%)
Influenza/Mycoplasma/COVID-19 (*n*, %)	(4, 20%)
Fever (*n*, %)	(20, 100%)
Shivering (*n*, %)	(11, 55%)
Dizziness and headache (*n*, %)	(13, 65%)
WBC (×10^9^/L)	7.68 (5.83, 9.05)
PLT (×10^9^/L)	129.55 ± 54.59
CRP (mmol/L)	80.15 ± 35.33
PCT (mg/mL)	0.53 (0.31, 1.12)
AST (U/L)	115.00 (88.00, 185.00)
ALT (U/L)	106.00 (73.00, 227.00)
Cr (μmol/L)	79.95 ± 35.70
GFR (μmol/L)	83.45 ± 23.33
PT (s)	12.10 ± 1.26
APTT (s)	30.45 (27.48, 34.60)
TT (s)	16.85 (16.25, 18.03)
INR	1.05 ± 0.12
DD (ng/mL)	3.71 ± 0.92

Note: WBC—white blood cell count; PLT—platelet count; CRP—C-reactive protein; PCT—procalcitonin; AST—aspartate aminotransferase; ALT—alanine aminotransferase; Cr—serum creatinine; GFR—glomerular filtration rate; PT—prothrombin time; APTT—activated partial thromboplastin time; TT—thrombin time; INR—international normalized ratio; DD—D-dimer.

**Table 2:** Number of samples collected at different time points and qPCR positivity rates.

Specimen Type	Before Doxycycline (*n*)	24 h After Treatment (*n*)	5 Days After Treatment (*n*)	Total (*n*)
Blood	20 (15/20, 75.00%)	20 (0/20, 0.00%)	20 (0/20, 0.00%)	60
NPS	20 (5/20, 25.00%)	20 (3/20, 15.00%)	20 (0/20, 0.00%)	60
Urine	20 (0/20, 0.00%)	20 (0/20, 0.00%)	20 (0/20, 0.00%)	60
Stool	16 (0/16, 0.00%)	4 (0/4, 0.00%)	0	20

**Table 3:** Analysis of nasopharyngeal swabs in scrub typhus patients (Ct values).

Patients	Time Points	GroEL	56 kDa	47 kDa
A1	Before doxycycline	31.23	32.94	30.16
A1	24 h after treatment	35.78	36.96	33.48
A2	Before doxycycline	33.81	36.95	33.06
A3	Before doxycycline	35.80	39.23	33.91
A4	Before doxycycline	33.16	36.47	34.50
A4	24 h after treatment	34.13	38.18	35.39
A5	24 h after treatment	35.30	40.31	35.27
A6	Before doxycycline	37.36	39.21	36.30

**Table 4:** TSA56 gene Ct values and qPCR quantification of *Orientia tsutsugamushi* in blood and NPS from scrub typhus patients pre-doxycycline.

Patient	Sample	Number of Sequences	qPCR		
			CT	CQ	SQ
A1	Blood	WCM-241102BYSZ-A7	29.55	30.59	7.37 × 10^3^
A1	NPS	WCM-241102XY	32.94	33.91	7.36 × 10^2^
A2	Blood	ZXZ-241204BYSZ-A23	32.83	32.61	1.81 × 10^3^
A2	NPS	ZSZ-241204XY	36.95	39.82	1.23 × 10^1^
A3	Blood	WCD-241206BYSZ-A26	30.68	30.28	9.15 × 10^3^
A3	NPS	WCD-241205XY	39.23	36.78	1.00 × 10^1^

Note: CT/CQ is the cycle number at which the fluorescence signal reaches the threshold value; SQ is the curve established by standards with known concentrations.

## Data Availability

All sequences analyzed during this study are available from the NCBI database (GenBank accession no. PX714883-PX714900).

## Supplementary Materials

The following supporting information can be downloaded at https://www.mdpi.com/article/10.3390/pathogens15020158/s1, S1 Text: Case definition; Table S1: Information on all reference sequences.

## References

[1] Taylor A.J., Paris D.H., Newton P.N. (2015). A systematic review of mortality from untreated scrub typhus (*Orientia tsutsugamushi*). PLoS Negl. Trop. Dis..

[2] Hazra S., Dutta A.P., Moitra S., Das S., Mallick S.K., Sarkar A., Binita P., Ayan C., Debapriya B., Arnab S. (2025). Seroprevalence of scrub typhus among patients with acute febrile illness. J. Fam. Med. Prim. Care.

[3] Luce-Fedrow A., Lehman M.L., Kelly D.J., Mullins K., Maina A.N., Stewart R.L., Ge H., John H.S., Jiang J., Richards A.L. (2018). A Review of Scrub Typhus (*Orientia tsutsugamushi* and Related Organisms): Then, Now, and Tomorrow. Trop. Med. Infect. Dis..

[4] Yue Y., Ren D., Liu X., Wang Y., Liu Q., Li G. (2019). Spatiotemporal patterns of scrub typhus in mainland China, 2006–2017. PLoS Negl. Trop. Dis..

[5] Jiang J., Richards A.L. (2018). Scrub Typhus: No Longer Restricted to the Tsutsugamushi Triangle. Trop. Med. Infect. Dis..

[6] Liu Q., Ji H., Shang M. (2025). Notified Vector-Borne Diseases—China, 2005–2024. China CDC Wkly..

[7] Ono A., Nakamura K., Higuchi S., Miwa Y., Nakamura K., Tsunoda T., Kuwabara H., Furuya Y., Dobashi K., Mori M. (2002). Successful diagnosis using scab for PCR specimen in Tsutsugamushi disease. Intern. Med..

[8] Jiang T., Jiang F., Qi W., Gu L., Wang L., Sun L. (2024). Expert consensus on the clinical diagnosis and treatment of scrub typhus. Chin. J. Zoonoses.

[9] Wang S., Huang J., Su J., Xi Y., Wang Y., Li M. (2007). Study on the characteristics of Tsutsugamushi disease in the epidemic areas of south islands in China. Zhonghua Liu Xing Bing Xue Za Zhi.

[10] Wang G., Fu R., Zhang L., Xue L., Al-Mahdi A.Y., Xie X., Qin A., Tang C., Du J., Huang Y. (2023). Genomic bacterial load associated with bacterial genotypes and clinical characteristics in patients with scrub typhus in Hainan Island, Southern China. PLoS Negl. Trop. Dis..

[11] Chen C., Chen T., Xue D., Liu M., Zheng B. (2025). Antibiotics in the treatment of scrub typhus: A network meta-analysis and cost-effectiveness analysis. J. Infect. Dev. Ctries..

[12] Gupta N., Boodman C., Jouego C.G., Broucke S.V.D. (2023). Doxycycline vs. azithromycin in patients with scrub typhus: A systematic review of literature and meta-analysis. BMC Infect. Dis..

[13] Kadosaka T., Kimura E. (2003). Electron microscopic observations of *Orientia tsutsugamushi* in salivary gland cells of naturally infected *Leptotrombidium pallidum* larvae during feeding. Microbiol. Immunol..

[14] Keller C.A., Hauptmann M., Kolbaum J., Gharaibeh M., Neumann M., Glatzel M., Fleischer B. (2014). Dissemination of *Orientia tsutsugamushi* and inflammatory responses in a murine model of scrub typhus. PLoS Negl. Trop. Dis..

[15] Trent B., Liang Y., Xing Y., Esqueda M., Wei Y., Cho N.-H., Kim H.-I., Kim Y.-S., Shelite T.R., Cai J. (2020). Polarized lung inflammation and Tie2/angiopoietin-mediated endothelial dysfunction during severe *Orientia tsutsugamushi* infection. PLoS Negl. Trop. Dis..

[16] Wang Z., Piao Y., An Z., Piao H. (2025). Pneumonia due to scrub typhus infection: A case report. BMC Pulm. Med..

[17] Moron C.G., Popov V.L., Feng H.M., Wear D., Walker D.H. (2001). Identification of the target cells of *Orientia tsutsugamushi* in human cases of scrub typhus. Mod. Pathol..

[18] Kelly D.J., Fuerst P.A., Ching W.M., Richards A.L. (2009). Scrub typhus: The geographic distribution of phenotypic and genotypic variants of *Orientia tsutsugamushi*. Clin. Infect. Dis..

[19] Duong V., Mai T.T., Blasdell K., Lo L.V., Morvan C., Lay S., Anukool W., Wongprompitak P., Suputtamongkol Y., Laurent D. (2013). Molecular epidemiology of *Orientia tsutsugamushi* in Cambodia and Central Vietnam reveals a broad region-wide genetic diversity. Infect. Genet. Evol..

[20] Varghese G.M., Janardhanan J., Mahajan S.K., Tariang D., Trowbridge P., Prakash J.A., David T., Sathendra S., Abraham O. (2015). Molecular epidemiology and genetic diversity of *Orientia tsutsugamushi* from patients with scrub typhus in 3 regions of India. Emerg. Infect. Dis..

[21] Long J., Zeng Z., Chen H., Tao X., Wu X., Chen S., Fang L., Zhang X., Xu J., Zhang L. (2024). Correlation between genotypes of *Orientia tsutsugamushi* and clinical characteristics of patients with scrub typhus in Guangzhou, China. Asian Pac. J. Trop. Med..

[22] Tian J.-W., Kong Y.-C., Han P.-Y., Xu F.-H., Yang W.-H., Zhang Y.-Z. (2024). Molecular epidemiological study of scrub typhus in residence, farm and forest habitats from Yunnan Province, China. PLoS ONE.

[23] Zheng L., Bi Z., Kou Z., Yang H., Zhang L., Zhao Z. (2015). Genotype diversity and distribution of *Orientia tsutsugamushi* in scrub typhus patients and rodents in Shandong, northern China. Infect. Genet. Evol..

[24] Kim S.W., Kim C.M., Kim D.M., Yun N.R., Neupane G.P., Pyun S.-H., Yu B.J. (2021). *Orientia tsutsugamushi* DNA load and genotypes in blood as a marker of severity. Acta Trop..

[25] Díaz F.E., Abarca K., Kalergis A.M. (2018). An Update on Host-Interplay and Modulation of Immune Responses during *Orientia tsutsugamushi* Infection. Clin. Microbiol. Rev..

