# Supplementary material for: Nasopharyngeal Swabs for Orientia tsutsugamushi Detection in Doxycycline Treatment: A Prospective Cohort Study in Hainan, China
Source: Pathogens. 2026 Feb 2;15(2):158. doi: 10.3390/pathogens15020158 (PMC12942918; doi:10.3390/pathogens15020158)
Supplement: Supplementary file 1 [file pathogens-15-00158-s001.zip › S1 Text.pdf]

S1 Text

Case definition

1 Epidemiological history: During the epidemic season, the patient had been in or been to the epidemic area of scrub typhus 3 weeks before the onset of the disease and had a history of field activities and sitting and lying in grassland.

2 Clinical manifestations:

2.1 A person had clinical symptoms of fever;

2.2 A person had lymphadenopathy;

2.3 A person had skin rash;

2.4 A person had specific eschar or ulcers.

3 Laboratory tests:

3.1 Single serum OX-K agglutination titer ≥ 1:160 in the Weil-Felix test;

3.2 Indirect fluorescent antibody immunoglobulin M (IFA IgM) ≥ 12800 or a fourfold or greater increase in the IgM titer between paired serum samples;

3.3 At least 2 positive PCRs targeting 56-kDa type-specific antigen (TSA), 47-kDa antigen, and groEL of the target genes;

3.4 Isolation of tsutsugamushi pathogens from clinical samples;

3.5 Detection of IgM positivity in the colloidal gold immunochromatographic assay (GIGA)

The diagnostic criteria for suspected cases meet 1, 2.1 plus 2.2 or 2.3; or who had no clear epidemiological history and who met criteria 2.1, 2.2 or 2.3 during the epidemic season.

The diagnostic criteria for clinically diagnosed cases meet suspected cases plus 2.4; or both 1, 2.1, and 2.4.

The diagnostic criteria for confirmed cases meet suspected cases plus 3.2 or 3.3 and 3.5; or meet clinically diagnosed cases plus any one of 3.
